# Efhamni: A Deep Learning-Based Saudi Sign Language Recognition Application

**DOI:** 10.3390/s24103112

**Published:** 2024-05-14

**Authors:** Lama Al Khuzayem, Suha Shafi, Safia Aljahdali, Rawan Alkhamesie, Ohoud Alzamzami

**Affiliations:** Department of Computer Science, Faculty of Computing and Information Technology, King Abdulaziz University, Jeddah 21589, Saudi Arabia; suhaashafii@gmail.com (S.S.); safiaaljahdali11@gmail.com (S.A.); ralkhamesie@gmail.com (R.A.)

**Keywords:** sign language recognition, deep learning, saudi sign language, CNN, pose estimation, MobileNet

## Abstract

Deaf and hard-of-hearing people mainly communicate using sign language, which is a set of signs made using hand gestures combined with facial expressions to make meaningful and complete sentences. The problem that faces deaf and hard-of-hearing people is the lack of automatic tools that translate sign languages into written or spoken text, which has led to a communication gap between them and their communities. Most state-of-the-art vision-based sign language recognition approaches focus on translating non-Arabic sign languages, with few targeting the Arabic Sign Language (ArSL) and even fewer targeting the Saudi Sign Language (SSL). This paper proposes a mobile application that helps deaf and hard-of-hearing people in Saudi Arabia to communicate efficiently with their communities. The prototype is an Android-based mobile application that applies deep learning techniques to translate isolated SSL to text and audio and includes unique features that are not available in other related applications targeting ArSL. The proposed approach, when evaluated on a comprehensive dataset, has demonstrated its effectiveness by outperforming several state-of-the-art approaches and producing results that are comparable to these approaches. Moreover, testing the prototype on several deaf and hard-of-hearing users, in addition to hearing users, proved its usefulness. In the future, we aim to improve the accuracy of the model and enrich the application with more features.

## 1. Introduction

Sign language is a visual language that uses hand gestures and facial expressions by people who have hearing problems. A total of 430 million people of the world’s population are in need of hearing rehabilitation, and 34 million of these are children [1]. Moreover, it is estimated that by 2050, around 2.5 billion individuals will have some form of hearing loss, and at least 700 million of them would need hearing rehabilitation [1]. In Saudi Arabia, as the General Authority for Statistics stated, the number of people with hearing problems has reached 59 thousand, including 12 thousand children in 2018 [2]. Deaf and hard-of-hearing people depend on sign language to communicate with each other, but there are more than 300 sign languages globally and more than one Arabic Sign Language (ArSL). In Saudi Arabia itself, the sign language slightly differs from one region to another [3]. Unfortunately, sign language is usually confined to the deaf and hard-of-hearing community and their relatives because people may not be familiar with the sign language, resulting in limited communication with the rest of the people and ignoring their right to equal educational and career opportunities.

Sign language recognition systems can be classified into two categories: device-based and vision-based [3]. Device-based recognition systems exploit wearable technology for hand gesture tracking, such as hand gloves, Kinect by Microsoft, and leap motion sensors. Most of the available solutions exploit wearable devices which are pricey and inconvenient for everyday use [4]. On the contrary, vision-based systems exploit computer vision and machine learning techniques to recognize hand gestures from images or video. These systems are easy to use for deaf and hard-of-hearing people since they only need to provide images or videos of them signing and do not require using any additional devices or sensors which might be costly, bulky, and uncomfortable to wear [4].

Additionally, sign language recognition systems can be classified into either continuous or isolated. In isolated sign language translation systems, only one sign gesture is displayed and translated at a time, while continuous sign language translation systems offer a complete clause translation [5,6]. While isolated sign language translation systems do not support complete sentences, they can aid in improving technologies for recognizing and translating individual signs, which is necessary for building more complex systems in the future that can handle phrases and sentences.

In recent years, with the revolution of technology, there has been increased attention to close the communication gap between the deaf and hard-of-hearing community and the rest of the people [7,8]. Despite the significant practical potential of sign language recognition, the problem of effectively recognizing sign language remains an open area of research due to its big challenges, which include differences in the semantic–syntactic structure of written and sign languages, internal factors (e.g., individual performance characteristics, context dependency), and external factors (e.g., lighting, background, shooting angle, etc.). As a result, there are currently no fully automated models and methods for recognizing several static and, most importantly, dynamic gestures. To develop such automated models, deep semantic analysis is required, which is challenging due to the limited number and the small size of sign language datasets [5]. Few research studies, however, have targeted the ArSL due to its complexity compared to the non-Arabic SL and the data unavailability (e.g., [9]), and even fewer have targeted the Saudi Sign Language (SSL) (e.g., [3,10,11,12,13,14,15]).

Despite the efforts by the Saudi Association for Deaf and Hard-of-hearing [16] to unify the Saudi Sign Language, Saudi deaf and hard-of-hearing people face difficulties in fully integrating with society and communicating with various social groups. This is due to the lack or scarcity of tools that translate the SSL into spoken or written Arabic and vice versa. This leads to isolation of the Saudi deaf and hard-of-hearing community from the hearing community.

Translating the SSL requires a special interpreter that works in between the hearing and deaf or hard-of-hearing person, which is an expensive and impractical method that interrupts the privacy of the conversation. Most available mobile applications that perform SSL translation (q.v. Section 2.4) rely on the availability of a human interpreter by booking appointments in advance. Thus, having a more efficient way of communicating is crucial for the overall social life of the deaf and hard-of-hearing person.

In this paper, we propose a mobile application, named Efhamni (an Arabic word that means “Understand me” in English), that helps Saudi deaf and hard-of-hearing people to communicate easily with others using sign language. In contrast to existing applications, Efhamni performs an automatic SSL translation to Arabic text and audio in an online direct manner using a deep learning model without the need for a human interpreter. Specifically, the application provides an isolated SSL recognition and translation system that exploits computer vision techniques, in which a deaf and hard-of-hearing person can record/upload a short video performed in SSL, and this video will then be translated to text, with the option of hearing the audio of the translated text. The deep learning model in this study is designed to recognize isolated dynamic signs rather than entire phrases due to the unavailability of continuous SSL datasets.

To the best of our knowledge, Efhamni is the first mobile application that provides a written and spoken translation for the SSL from a recorded/uploaded video. In addition, the application allows registered users to chat. For instance, a deaf and hard-of-hearing user can record/upload a video and send the translation to a non-deaf or hard-of-hearing person, who can reply by typing text in the chat. Moreover, the videos can be saved in the user’s library for future use and retrieval.

The rest of the paper is organized as follows. Section 2 reviews state-of-the-art research studies in the area, and Section 3 provides an overview of the system. Section 4 illustrates the data gathering methods and the analysis of the collected data. Section 5 discusses the system design and Section 6 presents the implementation of the proposed mobile application. Section 7 discusses the development of the Saudi Sign Language Translation model using deep learning. Section 8 presents the functional and usability testing of the proposed system. Finally, Section 9 presents the discussion and Section 10 concludes our paper.

## 2. Related Work

Sign language translation approaches can be classified into three categories based on the direction of translation. The first category includes approaches that translate between the sign language and the text or audio in both directions. Only a few approaches fall into this category, such as the work of Faisal et al. [12] and Shinde et al. [17]. The second category includes approaches that generate sign languages from text or audio. Most of the proposed approaches in the literature fall into the second category because of their simplicity (e.g., [18,19,20,21]). The last category includes approaches that translate from sign language to text or audio. Translating from sign language to text or audio is more challenging than translating from text or audio to sign language because it requires significant processing of visual cues, which entails some difficulties. These difficulties include identifying the beginning and the end of the sign considering that some signs are short and others are long, the varying speed at which the sign language is performed by the deaf person, and the use of emotions accompanied with the sign language [22]. In the following subsections, we review state-of-the-art datasets, related approaches, and applications.

### 2.1. Sign Language Translation Datasets

A list of sign language datasets utilized in earlier studies is included in Table 1. These datasets include the following:

1. The Turkish Sign Language (AUTSL) [23] dataset is a large-scale, multimode dataset that contains isolated Turkish sign videos. It contains 226 signs that are performed by 43 different signers. The total number of video samples is 38,336.
2. The Word-Level American Sign Language (WLASL) [24] dataset contains 34,404 videos of 3126 common different words performed by 119 signers.
3. The HAnd Gesture Recognition Image Dataset (HaGRID) [25] contains 554,800 images divided into 18 classes of gestures (in addition, some images have no_gesture class if there is a second free hand in the frame). Samples were recorded by 37,583 signers.
4. The Russian Sign Language (TheRuSLan) [26] dataset is a record of 164 gestures performed by 13 signers with at least five iterations.
5. The Russian Sign Language (SLOVO) [27] dataset contains 20,400 videos for 1000 sign language gestures from 194 signers.
6. The Arabic Sign Language (ArSL) [28] dataset contains 3450 samples of 23 gestures performed by three participants. Each subject was asked to repeat the gestures 50 times. Therefore, there are 150 samples in the dataset for each gesture.
7. The King Saud University Saudi Sign Language (KSU-SSL) [10] dataset includes 16,000 videos of 40 words, in addition to the alphabet and numbers performed by 40 signers.

Datasets 1–5 are out of this paper’s scope, which is developing an application that translates SSL to text and audio. These datasets target particular countries’ sign languages, such as American [24], Turkish [23], and Russian [26,27]. In addition, many Arabic sign language datasets are not accessible to the public or contain only a small number of signs or signers, such as the ArSL [28] dataset, which contains 23 signs performed by three signers. Due to the fact that sign language varies from country to country, the KSU-SSL dataset [10] is the chosen dataset for our application, Efhamni, since it targets SSL and includes a reasonable number of signs.

**Figure 1:**
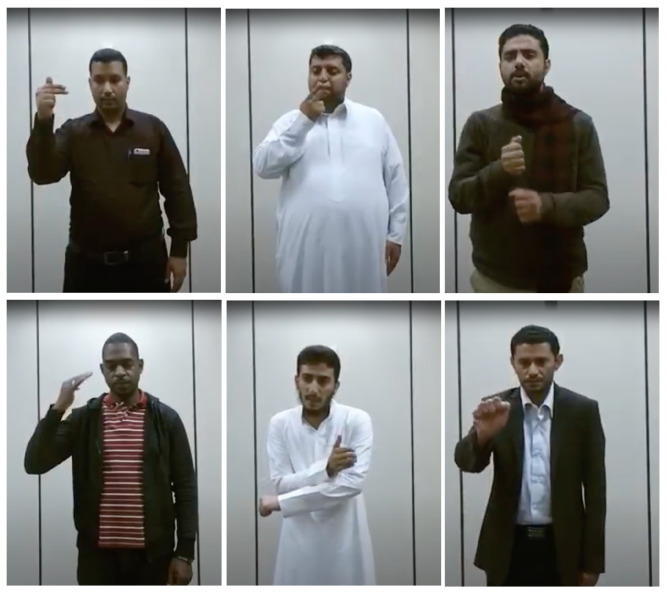
Examples from the KSU-SSL dataset [10].

The KSU-SSL dataset [10] includes chosen gestures from commonly used Saudi sign language words and expressions. These expressions include both single-handed and two-handed signs. The KSU-SSL dataset was created with the help of 40 people. The participants were a mix of hard-of-hearing and hearing people. During five different sessions, each participant was instructed to execute each sign five times. This dataset was recorded using various technologies, including RGB cameras and the Microsoft Kinect. The dataset recording sessions were conducted in an unstructured setting with no constraints. There were no restrictions on the participants’ clothes, lighting, or background color. Examples of images taken from the KSU-SSL dataset are shown in Figure 1. The KSU-SSL dataset has been used in several other approaches, such as [11,14,15].

### 2.2. Translation of Non-ArSL to Text or Audio

Novopoltsev et al. [29] proposed fine-tuning models that work on CPU in real time by testing two models, Video Swin Transformer and Multi-scale Vision Transformer (MViT), which were pre-trained on the Kinetics600 dataset. Then, the models were fine-tuned on three different sign language datasets: WLASL, AUTSL, and RSL. The authors fine-tuned the Kinetics pre-trained network using different experiments, and concluded that incorporating datasets from other sign languages significantly improved the ability to recognize sign gestures. Specifically, the MViT model, when trained on RSL then AUTSL, achieved an accuracy of 95.72%.

Ryumin et al. [30] proposed an Audio-visual Speech Recognition (AVSR) and gesture recognition methods and models. This study introduces two deep neural network-based model architectures: one for AVSR and one for gesture recognition. The main novelty regarding audio-visual speech recognition lies in fine-tuning strategies for both visual and acoustic features and in the proposed end-to-end model, which considers three modality fusion approaches: prediction level, feature level, and model level. The main novelty in gesture recognition lies in a unique set of spatial–temporal features, including those that consider lip articulation information. The study achieved an accuracy of 98.56% for the AUTSL dataset.

Ryumin et al. [31] proposed a novel approach for sign language recognition that utilizes transfer learning to improve the recognition rates on a target sign language corpus. The approach is divided into two steps: first training the model on the AUTSL and TheRusLan datasets for simultaneous gesture recognition, then fine-tuning the pre-trained model on the target corpus using transfer learning. The approach achieved an accuracy of 93.38%.

Hrúz et al. [32] proposed a comprehensive study of appearance- and pose-based methods which were applied on two well-known datasets, AUTSL and WLASL300. The study used a neural ensemble utilizing the Covariance Matrix Adaptation Evolution Strategy (CMA-ES) method, which estimates optimal weighting parameters of an ensemble. The study achieved an accuracy of 96.37% on the AUTSL dataset and 73.87% on the WLASL300 dataset.

De Coster et al. [33] proposed an isolated sign language recognition from RGB videos using Pose Flow and Self-Attention. Specifically, they trained a Video Transformer Network (VTN) which includes extracting human pose key points, using OpenPose, to capture the body movement and hand cropping to capture different hand shapes. The approach achieved an accuracy of 92.92% on the AUTSL dataset.

Jiang et al. [34] proposed a Sign Language Graph Convolution Network (SL-GCN) to extract information from the whole-body skeleton graph. The study utilized Separable Spatial–Temporal Convolution Network (SSTCN) to exploit the whole-body skeleton features, which significantly improved the accuracy of detecting whole-body key points. In addition, a Skeleton Aware Multi-modal SLR (SAM-SLR) framework for RGB and RGB-D based Sign Language Recognition (SLR) was used, which learns from six modalities and achieves state-of-the-art performance on the AUTSL dataset. The study achieved the highest performance in both RGB (98.42%) and RGB-D (98.53%).

Sincan et al. [23] presented a new large-scale isolated Turkish Sign Language dataset, named AUTSL, and provided multiple deep learning-based models that are based on a vanilla CNN + LSTM architecture and trained on RGB-D and RGB data. RGB-D data combined with the CNN + (Feature Pooling Module) FPM + BLSTM + Attention architecture achieved the best result on RGB-D of 62.02%.

Sabeenian et al. [35] proposed an American Sign Language (ASL) recognition system that translates gestures to text. The proposed approach used a custom CNN (Convolutional Neural Network) model which consists of 11 layers. Initially, the RGB images were extracted from the video recorded with Webcam. Then, the images were converted to grayscale images or HSV (hue, saturation, value) plane. Subsequently, the images were re-scaled to the size of the trained images. After that, the pre-trained custom CNN model was applied and the hand gesture predictions were made. Finally, a SoftMax layer was applied before producing the output. The model was trained on an ASL dataset that consists of 27,455 training samples with 784 features. In addition, the model was validated using 7172 images, and the validation accuracy of the model was greater than 93%. However, the cluttered background in the extracted images affected the accuracy of the model. Nonetheless, the model gave better accuracy using the Open-CV (Open-Source Computer Vision Library) with ROI (region of interest) for extracting hand gesture frames.

### 2.3. Translation of SSL or ArSL to Text or Audio

Faisal et al. [12] developed a system called the Saudi Deaf Companion System (SDCS), which facilitates communication from the deaf to the hearing and vice versa. The system includes a module called the Sign Recognition Module (SRM), which is responsible for recognizing the signs made by deaf individuals and converting them into text. To achieve efficient performance and compactness within the suggested SRM, a self-developed 3D Graph Convolutional Network (3DGCN) with a small number of trainable parameters was used. The system is designed to be integrated into portable electronic devices like laptops, tablets, or mobile phones, facilitating easy and accessible communication. In addition, the paper discusses the development of a comprehensive Saudi sign language database, KSU-SSL, which includes videos of 293 Saudi signs across various domains and performed by 33 signers [13].

Bencherif et al. [11] developed an automatic Arabic Sign Language (ArSL) recognition system using the concatenation of a 3DCNN skeleton network and a 2D point convolution network. The study used its own ArSL video dataset, which contains 80 static and dynamic signs repeated five times, resulting in 400 signs, which include the Arabic alphabet, numbers, and some daily use signs. The proposed approach feeds video files to the OPL network to generate the hands and body key points. Each RGB frame is thus converted to a set of 2D key points (2DCNN network for key point estimation in the body). Then, the system collects a small sequence of frames and sends them to the Skeleton CNN Network (SKN) to generate the recognized Arabic sign. The system was evaluated on the static and dynamic signs in signer-dependent and signer-independent modes and the achieved accuracies were 89.62% and 88.09%, respectively, when the signs were mixed. Similar to the approach of Al-Hammadi et al., the approach of Bencherif et al. was not implemented as a mobile application and only considers translating sign language to text.

Al-Obodi et al. [3] proposed a Saudi Sign Language (SSL) text recognition system using a deep Convolutional Neural Network (CNN). The proposed system was trained on a constructed dataset that includes 27,301 static images for 40 Saudi signs containing the alphabet, numbers, and one word described by one gesture of “Me” or “I” (40 classes). The results show that the system achieved over 97% accuracy for training and over 99% accuracy for testing. The proposed system was developed in two versions: a desktop application and a mobile application, for ease of use. However, the study is limited by the categories of gestures, which consist of only alphabets, numbers, and one word. Moreover, it provides translation from hand gesture images to text only.

Al-Hammadi et al. [10] proposed an automatic hand gesture recognition system using 3D Convolutional Neural Network (3DCNN) for spatiotemporal feature learning from videos, based on two approaches. The first approach is a single 3DCNN structure (one instance of the 3DCNN) trained to extract the hand gesture features from the entire video through three phases: video pre-processing, feature learning, and classification. The second approach is a parallel 3DCNN structure (three instances of the 3DCNN) trained to extract the hand gesture features from the beginning, middle, and end of the video sample. Then, fused region-based features were extracted using three feature fusion techniques, which are multilayer perception (MLP) neural network, long short-term memory (LSTM) network, and stacked autoencoder. In both approaches, the final classification phase involves feeding the extracted features into a SoftMax layer, which outputs classes with maximum probabilities. The two approaches were evaluated in two modes, signer-dependent and signer-independent, using three different hand gesture datasets, namely the King Saud University Saudi Sign Language (KSU-SSL) dataset, the Arabic Sign Language (ArSL) dataset, and the Purdue RVL-SLLL American Sign Language dataset. The approach’s accuracy rates for the signer-dependent mode were much higher (98.12%, 100%, and 76.67% for the three datasets, respectively) than the signer-independent mode (84.38%, 34.9%, and 70% for the three datasets, respectively). While Al-Hammadi et al.’s approach covers more words than Al-Obodi et al.’s approach, the accuracy rates for its signer-independent mode are low, especially for the ArSL dataset. Moreover, the approach was not implemented as a mobile application for ease of use.

Table 2 presents a comparison of the proposed approaches for translation of non-ArSL and ArSL/SSL to text or audio in terms of the publication year, dataset, approach, system type, accuracy, and language.

### 2.4. Sign Language Translation Applications

Available existing applications that target the sign language translation problem in different languages and for different countries are shown in Table 3, which compares the available applications in terms of the target language, scope, platform, accuracy, speed, and features.

## 3. System Overview

**Figure 2:**
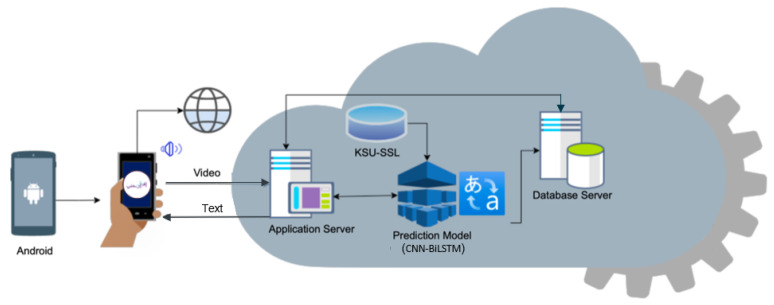
Efhamni’s architecture.

The high-level architecture of the Efhamni mobile application is depicted in Figure 2, in which the system’s input, cloud processing, and output are identified. The SSL recognition system is implemented on an Android environment and needs an internet connection.

The proposed application is deployed on a mobile device, giving its users the freedom of movement, which enables them to use the application anytime and anywhere. In addition, the application considers the users’ different hand shapes and sizes, as well as their various types and colors of clothes.

The system works as follows. A user either records or uploads an SSL video to the application. The application also enables users to edit the recorded or uploaded video to select a specific clip before sending it to the application server. At the server, the video will be pre-processed to extract key-frames with meaningful signs. Then, an edge detection method is applied to the frames to detect the hand gestures and then extract hand gestures using CNN to recognize and translate the signs to text.

A video of a person performing SSL will be the input to the system, and it will be translated to text and audio and the video will be saved in the database. Efhamni uses the Quick Response Code (QR Code) which is a two-dimensional version of the barcode to initiate a chat session between any two registered users. A QR Code has several advantages, including speeding up the communication process between members while preserving the registered users’ privacy. Users, whether deaf, hard-of-hearing, or hearing, can scan the QR Code to connect to a registered user’s device. For instance, a deaf or hard-of-hearing user scans the QR Code of a hearing person to open a chat session to which the translation of the video, in text and audio, will be sent. Upon receiving the translation, the hearing user can reply using text.

## 4. Data Gathering

Data gathering and analysis for the system design enable the construction of a credible and usable system that can serve its intended purpose. In this paper, two gathering methods, interviews and questionnaires, were used. The goal of each data collection method and how it is administered to participants are described in the following subsections.

### 4.1. Interviews

Three interviews were conducted with SSL experts. Each interview consists of 12 questions. The first five questions were related to the background of the interviewees, including their job, role in the deaf and hard-of-hearing community, the motive behind joining the community, expertise duration, and the number of sign languages that the interviewee had mastered. The next three questions are related to the SSL, including whether or not the language includes an equivalent translation for each spoken word, whether there are different dialects, and how much they vary. The last four questions are related to deaf and hard-of-hearing people, including the most common places in which they need translation, their level of proficiency in reading and writing, proficiency in using smart devices and mobile applications, and finally, the usefulness of having a mobile application that translates sign language videos to text and audio via an online direct translation. A summary of the interviewees’ responses and the main highlights of each interview are given below.

1. The first interviewee has been actively involved in sign language and the deaf and hard-of-hearing community for over 20 years. She established the first deaf and hard-of-hearing Women’s Club in Jeddah, Saudi Arabia, in 2001, then turned the club into an association, making it the first women’s deaf and hard-of-hearing association in Jeddah. She organized many educational events and activities for deaf and hard-of-hearing women and provided support and assistance concerning their education, marriage, and employment. The main highlights from the interview were that she thought our application was a practical idea and would serve many deaf and hard-of-hearing people since they are good at using smartphones and are accustomed to video communication. Also, sign language is their native language, so they will not find it challenging to film a short video of themselves to translate the gestures from it. The interviewee trained more than 850 people in the health sector in sign language. Thus, through her experience in the field, she advised us to choose vocabulary used in hospitals.
2. The second interviewee has been an educational supervisor in the Department of Education in Makkah for more than 27 years. He is a member of the SSL Interpreters Association who has served as a sign language interpreter at the General Presidency for the two Holy Mosques and a translator in government departments and courts. In addition, he trained security patrol personnel in sign language and volunteered to translate educational videos and lessons. The main highlights from the interview include the interviewee’s view of the application as essential and unique due to a lack of similar applications in the market. The interviewee recommended that we concentrate on the application’s deployment in hospitals and government sectors. In addition, he stressed the importance of having such an application given the high number of deaf and hard-of-hearing people in Saudi Arabia, which exceeded half a million people, and the limited number of certified translators, which does not exceed 102. Furthermore, the current applications that serve the deaf and hard-of-hearing community are inaccurate and do not provide automatic translation from sign language to text and audio. He mentioned that the proposed application will significantly serve the deaf and hard-of-hearing users and facilitate their communication with others as they go through their daily activities.
3. The third interviewee has been a mathematics teacher at a school for the deaf and hard-of-hearing for 22 years, with a master’s degree in special education. She is also a researcher interested in improving the communication between deaf or hard-of-hearing children and hearing people. She supported the idea of our application and emphasized its importance, especially since it aligns with her cause of integrating deaf and hard-of-hearing and hearing people, especially in schools. She stated that deaf and hard-of-hearing youth are good at using smartphones. The difference between the dialects in the SSL does not constitute an obstacle to communication, and if we use the vocabulary in the Saudi Sign Dictionary, it will be known to all dialects. In her experience, teaching deaf and hard-of-hearing people is a complicated process and takes time, and it is best accomplished by dividing them into small groups that do not exceed nine people per session. She indicated that the most crucial vocabulary that should be covered by the application is signs used in situations to ask for help when feeling tired or sick.

To summarize the main points taken from the interviews, deaf and hard-of-hearing people are good at using their smartphones. They are also used to recording themselves and performing sign gestures with only one hand. Thus, using our application would not be a problem for them. However, since sign language is considered their first language and they rarely understand English, Efhamni interfaces will be in Arabic. Nevertheless, all experts agreed that the Efhamni application is necessary and will help the deaf and hard-of-hearing community greatly and confirmed that no other applications exist that translate sign language to text and audio.

### 4.2. Questionnaire

Two questionnaires were used in this study, one for Saudi Sign Language experts and one for deaf and hard-of-hearing people. All questions in both questionnaires were written in English and Arabic. The questionnaires were reviewed by three experts in Saudi Sign Language and speech and language therapists to ensure that the questions were clear and easy to understand. Based on reviewing the first draft of the deaf and hard-of-hearing questionnaire, some of the questions were re-written in a simpler form to make them easier to understand for the deaf and hard-of-hearing people, as the written Arabic language is considered their second language, where their first language is sign language. In addition, open questions were replaced by multiple-choice questions, and the choices of some questions were modified according to the experts’ reviews.

The Google platform was used for creating and collecting the questionnaires’ responses. The questionnaires were distributed through X (formerly Twitter) and through organizing an event in cooperation with the Women’s Association for Deaf and Hard-of-hearing in Jeddah, Saudi Arabia. During the event, the purpose of the data collection and the idea of the application was explained to participants. An interpreter explained the purpose of the data collection and the questions to the deaf and hard-of-hearing particpnats to make sure that they understood the questions correctly and provided accurate responses.

A total of 10 responses were collected from Saudi Language Experts. The expert questionnaire and results of the collected responses are shown in Table 4. Most of the experts who participated in the sign language expert questionnaire were male with a percentage of 70%, and they were all 30 years old and above. Half of the experts (50%) hold a postgraduate degree in areas related to deaf and hard-of-hearing people’s education and therapy. All of the experts interact regularly with deaf and hard-of-hearing people, where 80% of them have experience of 8 years or more. According to the experts’ opinions, most of the deaf and hard-of-hearing people (80%), are proficient in using mobile phones and their applications. The communication method used the most by deaf and hard-of-hearing people inside and outside their homes is sign language. However, the experts pointed out that all deaf and hard-of-hearing people face difficulties when communicating with people in public places, which indicates the need for a supportive tool for sign language translation. The experts pointed out that hospitals (25%), government offices (25%), and malls (34%) are the places in which deaf and hard-of-hearing people face difficulties the most. It was also indicated that the inability to communicate with others is the biggest barrier for deaf and hard-of-hearing people. When asked about using translation applications, the experts indicated that only 40% of deaf and hard-of-hearing people use these kinds of applications, having Turjuman and Eshara as the most used ones, with percentages of 35% and 42%, respectively. More importantly, the experts believe that 70% of deaf and hard-of-hearing people are willing to use translation applications and they think that fast translation of sign language to text is the most important feature in such applications.

For the deaf and hard-of-hearing people questionnaire, a total of 110 responses were collected. The distributed version of the deaf and hard-of-hearing questionnaire along with collected responses are shown in Table 5. Most of the participants who participated in the deaf and hard-of-hearing questionnaire were female, with a percentage of 95%, and the majority were between 18 and 29 years old. Most of the participants (69%) hold either a high school diploma or a bachelor’s degree. All of the participants are either deaf or have hearing difficulties. Most of the participants have moderate experience (68%) in using mobile phones and their applications, mostly spending between 3 and 5 h (73%) using mobile phones. Most of the participants use sign language to communicate with others inside and outside their homes, followed by writing. However, they indicated that they mostly face difficulties when communicating with people in public places, which supports the idea of developing a tool for sign language translation. The participants pointed out that hospitals (39%), malls (16%), and restaurants (15%) are the places in which they face difficulties the most. Among the other places that the participants mentioned were parks and places of worship. It was also indicated that the inability to communicate with others is the biggest barrier for them. When asked about using translation applications, most of the participants (54%) have used mobile applications for sign language translation, having Eshara, Esharati, and Turjuman as the most used ones, with percentages of 30%, 27%, and 25%, respectively. The majority of participants (58%) are willing to use translation applications in public places and they think that ease of use is the most important feature in such applications.

The results can be summarized as follows. Most deaf and hard-of-hearing people are good at using technology, and will use a mobile application to translate sign language to audio and text, and they face difficulties in communicating outside the house, especially in hospitals.

## 5. System Design

The process of defining the components, modules, interfaces, and data for a system to meet given criteria is known as system design [37]. In the following subsections, the system design of Efhamni is presented using a use case diagram, sequence diagrams, and flowcharts.

### 5.1. Use Case Diagram

A use case is a usage scenario for a piece of software. It is commonly used to identify circumstances where a piece of software might be beneficial. A use case is a set of actions or event steps that describes how an actor and a system interact to achieve a goal.

**Figure 3:**
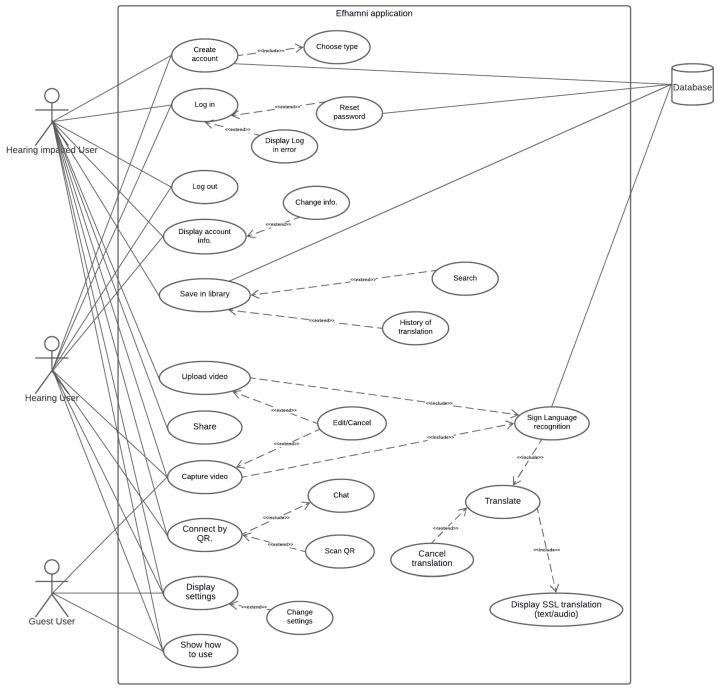
Efhamni (Understand Me) application use case.

The Efhamni use case diagram is shown in Figure 3. The use case diagram illustrates the application’s functional requirements, specifies the interactions of the users with the system though the different use cases, and shows the relationships between these use cases.

### 5.2. Sequence Diagram

**Figure 4:**
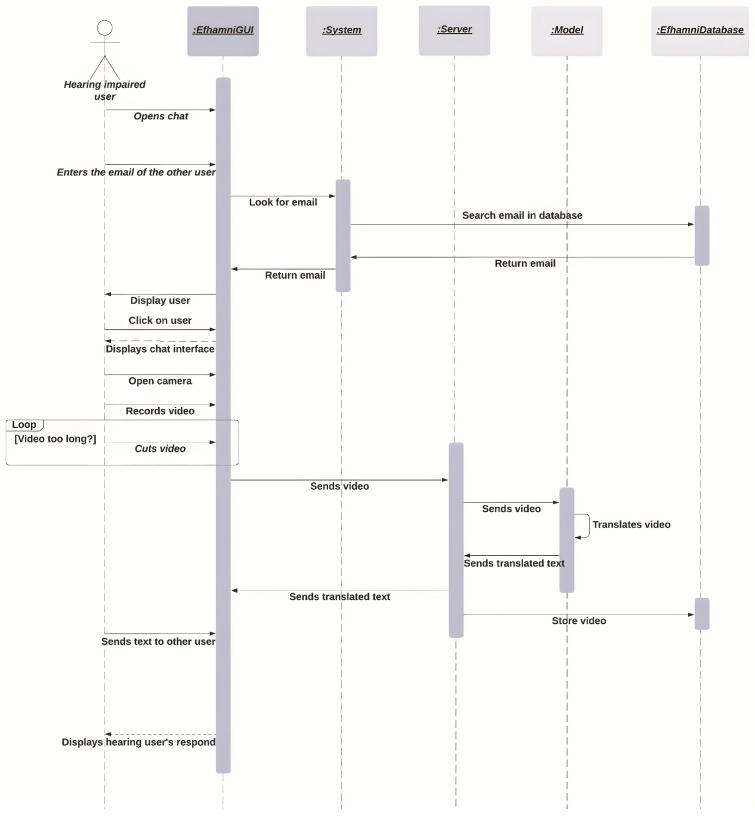
Deaf and hard-of-hearing chat sequence diagram.

Figure 4 illustrates the sequence diagram for the deaf and hard-of-hearing users when they start a new chat. The diagram starts with the deaf and hard-of-hearing user opening the chat icon and then either scanning another user’s QR code or searching for another user by his/her email. If the email is not found in the system, the user will not be able to chat with that person. If the email is found successfully, the user may press the “Open camera” button and start the translation process from SSL to text and audio. The process starts with the user opening the camera in the application, and then recording a limited-length video; if the video is too long, the user can clip it to fit the maximum length. Then the user presses the send button and the video is sent to the server to be processed, after which it is sent to the model for translation. The model then sends the translated text back to the server, which sends the translated text to the user’s interface, and the server stores the video in the database. The system then sends the text to the other user.

### 5.3. Flowchart

**Figure 5:**
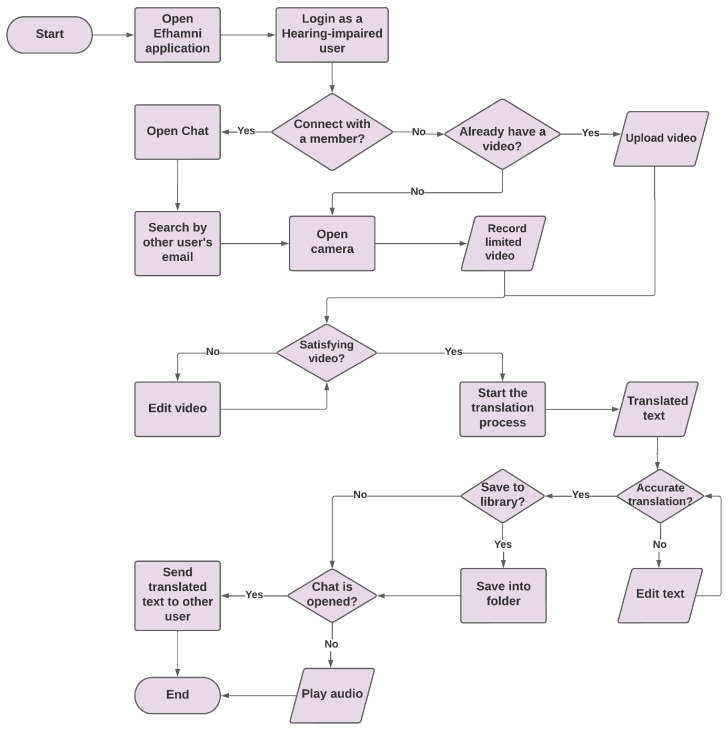
Deaf and hard-of-hearing flowchart.

Figure 5 presents the Efhamni SSL translation system. The flowchart starts by opening the Efhamni application, then logging in as a deaf and hard-of-hearing user, and after that asking if there is a member to connect with. If the answer is yes, then the user opens the chat and searches for the other user by QR code or email. If the answer is no to the previous question, then the user will be asked to upload or record a video. If the user does not have a video containing the user performing a sign gesture, and it was yes to the connect with a member question, it continues to the main function, which is opening the camera, then recording a limited-length video. If the answer is yes when asked if there is already a video containing the user performing a sign gesture question, then the user uploads the video and proceeds to see if the video is accurate. If not, the user can edit the video, and see if it is accurate. If yes, then the translation process starts, and the translated text is obtained. The user will then be asked if the translation is accurate, and if not, he/she can edit the text and see if it is accurate again. If the answer is yes, the user will then be asked if he/she wants to save the video in his/her library, if yes, it will be saved into the library’s folder, and if no, the user will be asked if there is a chat opened. If the answer is yes, the translated text will be sent to the other user, and if no, the audio may be played.

## 6. System Implementation

The development of the Efhamni application required various technologies, such as Flutter https://flutter.dev (accessed on 10 May 2024) for developing the front and back end of the application, Firebase https://firebase.google.com (accessed on 10 May 2024) for creating the database of our application, Google Colab Pro+ https://colab.research.google.com (accessed on 10 May 2024) for developing the deep learning model, and Flask https://flask.palletsprojects.com/en/2.1.x/ (accessed on 10 May 2024) for deploying the deep learning model and configuring the backend with the frontend.

The Efhamni application has three different types of users, which are deaf and hard-of-hearing, hearing, and guest users. There are common interfaces between all the users and there are special interfaces for each type of user.

**Figure 6:**
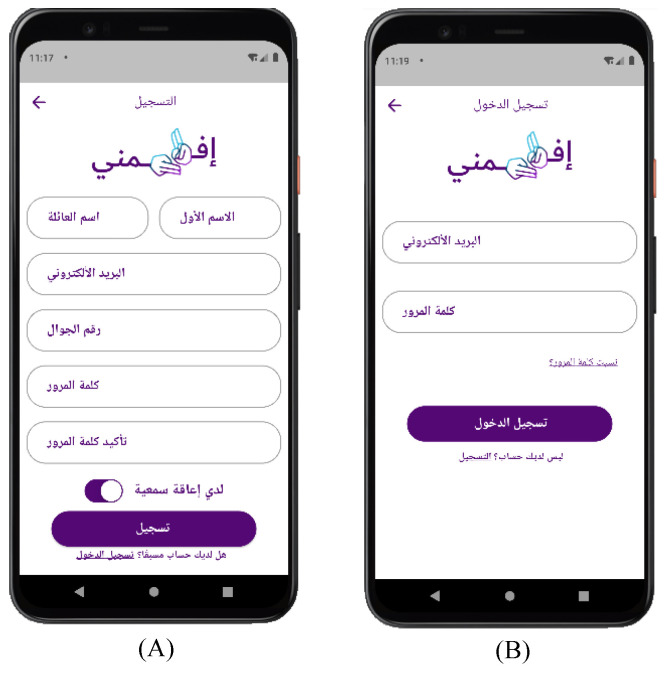
Efhamni (Understand Me) Application: (**A**) sign-in and (**B**) sign-up screens.

Figure 6 shows the sign-up and sign-in screen. Once the user clicks on “Sign Up”, the sign-up screen will appear. The user’s full name, e-mail, phone number, password, and type of user are required to create an account. Then, the registered user will be able to sign in to continue using the application. If the entered user information is correct, the user will be allowed to log in and will be directed to the home screen. Otherwise, the user will not be allowed to log in.

**Figure 7:**
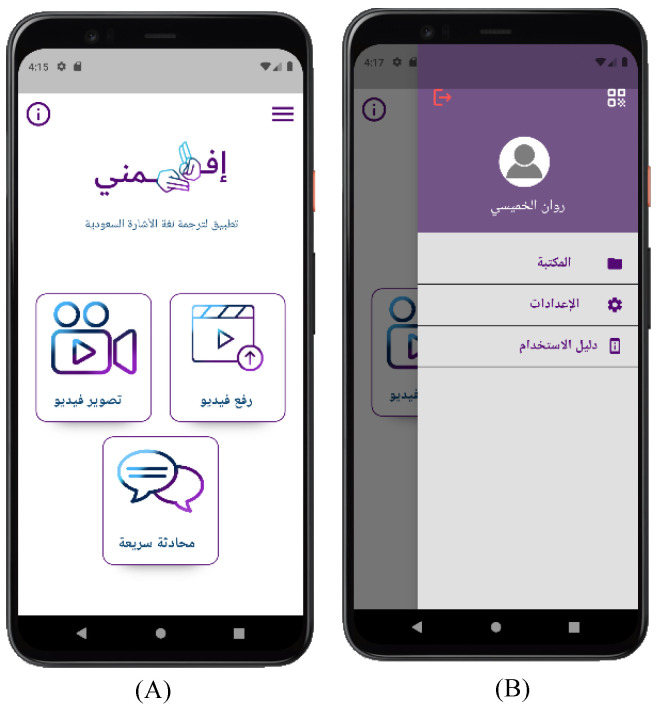
Efhamni (Understand Me) Application: (**A**) deaf and hard-of-hearing home page and (**B**) deaf and hard-of-hearing side menu screens.

In Figure 7, the home page screen (A) contains an option “video recording” to capture a limited-length video of two minutes and an option “upload video” to upload the video from the user device. It also contains a quick chat option to chat with registered users. If the deaf and hard-of-hearing user selects the menu icon, a side menu screen (B) will emerge, including the user library, settings, and instructions on how to use the app. It also contains the user’s QR and logout button.

**Figure 8:**
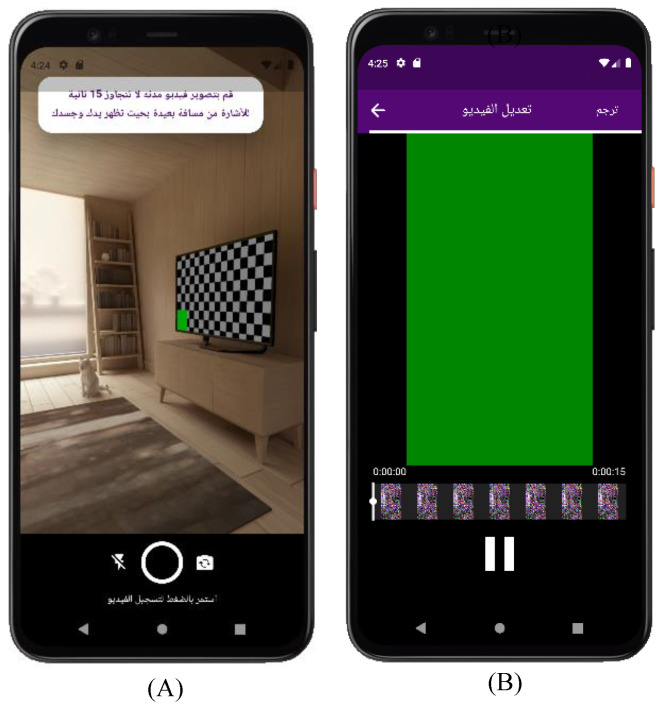
Efhamni (Understand Me) Application: (**A**) camera and (**B**) editing screens.

The camera and editing screen for Efhamni is shown in Figure 8. The camera screen (A) asks if the user wants to capture a video of no more than 15 s, and this feature is available to all users regardless of how they access the system. After that, the editing screen (B) will open for the user, through which he/she can edit the video and begin the translation process.

**Figure 9:**
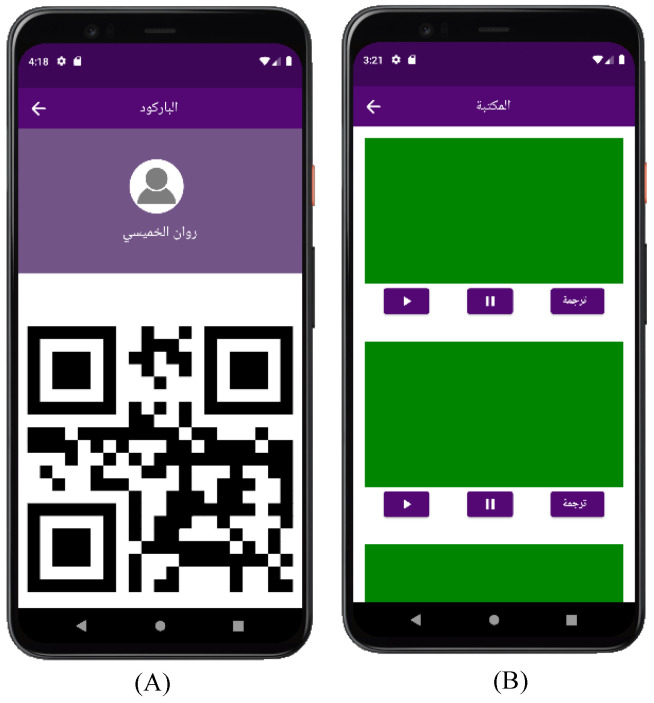
Efhamni (Understand Me) Application: (**A**) QR of the person enrolled in the system and (**B**) deaf and hard-of-hearing side menu screens.

Figure 9 shows the QR and library screen. For registered users, the screen in (A) displays the QR of the person enrolled in the system and is accessed through the side menu, by clicking on the QR icon in the upper right corner. Screen (B) shows the library where user videos are saved and is only for deaf and hard-of-hearing users.

**Figure 10:**
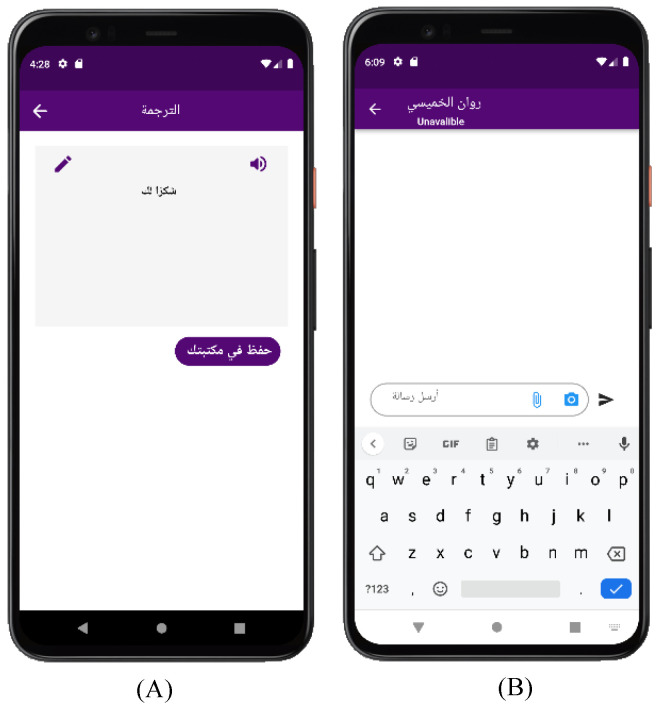
Efhamni (Understand Me) Application: (**A**) deaf and hard-of-hearing translation and (**B**) deaf and hard-of-hearing chat screens.

Figure 10 shows the translated videos interfaces. After translating a video into SSL, it will appear on the translation screen, where you can play the audio or modify the translation and also share or save it to your library. In screen (B), the chat screen appears, where you can click on the camera icon or the upload icon in the send bar at the bottom of the screen to open the camera or studio, after which the translation process will start, then send the translation as a message to the other party.

## 7. The Saudi Sign Language Translation Model

Our prediction model uses a Convolutional Neural Network (CNN) with a Bidirectional Long-Short Term Memory (BiLSTM) architecture. The advantage of using CNN is that it requires significantly less time in pre-processing since it can learn the characteristics of the data with proper training, as opposed to other primitive approaches that are hand-engineered. Moreover, BiLSTM is useful for translating SSL, since it considers sequences of input, which is useful for phrase cases. The basic CNN architecture takes photo frames as input, then goes through different layers to finally connect the layers and extract the output from them. The first set of layers are convolutional layers that apply filters on the frames to extract features, followed by pooling layers for reducing spatial size. Subsequently, the LSTM layers classify sequence frames to a phrase. The output from the LSTM layer is a three-dimensional matrix that must be flattened. Flattening means to unroll all its values into a vector. The flattened vector is then fed into fully connected layers to produce a higher-order feature representation of the different classes. Finally, the output layer produces the translated text. This text is then saved in the database and translated into Arabic text before being translated into audio.

The model was trained on the KSU-SSL dataset [10], which covers 40 words and the alphabet and numbers of the Arabic language, comprising 80 different signs. The dataset includes 16,000 recorded videos in an uncontrolled environment.

**Figure 11:**
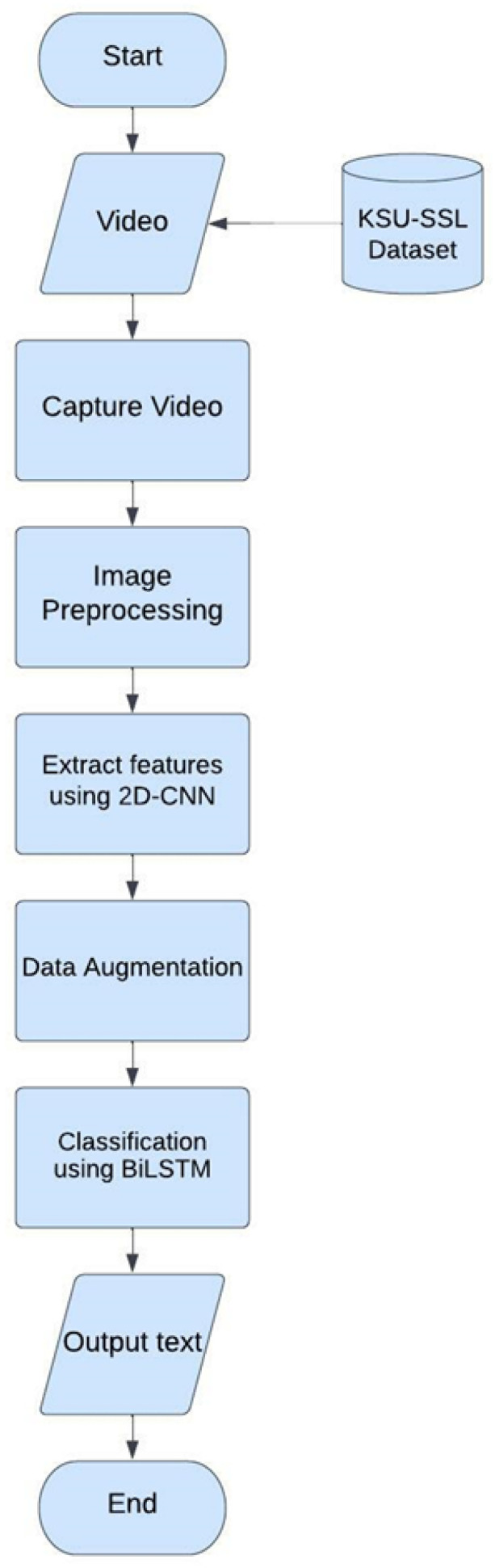
Flowchart of the deep learning model.

Figure 11 shows the flowchart of the proposed model and in what follows, we explain each of the mentioned steps.

### 7.1. Image Preprocessing

The VideoCapture method from the OpenCV library, which utilizes BlueGreenRed (BGR) instead of RedGreenBlue (RGB), is used to capture the frames of a video as BGR. Subsequently, a loop through all frames in a single video is performed to find the action magnitude over each frame in a video, by tracking the position of the object in the sequential frames. Our model takes a specified number of frames per video, which is 30, to classify the signs. The image preprocessing steps are as follows:

1. Convert the frame colors from BGR to gray.
2. Blurring the frames.
3. Since the background is stationary, we use background subtraction to subtract the unnecessary region.
4. The function that OpenCV provides for this purpose is cv2.absdiff, which is a function that helps in finding the absolute difference between the pixels of the two image arrays. Thus, it finds the best frames to extract, given a start frame.

### 7.2. Feature Extraction

In sign language, there are multiple factors that influence the classification of the sign. Therefore, to distinguish signs with different poses, estimating hand gesture, hand movement, and hand position are needed. An open-source human pose estimation framework, called MediaPipe, was used for feature extraction, which extracts key points as a skeleton using variant CNN models, such as MobileNetV2 for pose estimation, face detection, and Single-Short Detector (SSD) for hands. The MediaPipe is a holistic pipeline that integrates separate models for pose, face, and hand components, each of which is optimized for their particular domain. Specifically, the SSD uses bounding boxes (called anchors) for hand detection, where the parameters of these anchors could be adjusted to better fit the typical hand shapes and sizes in various configurations. The overall feature extraction steps are as follows:

1. Estimate the human pose with pose detector and subsequent landmark model, with the output as follows:
  - X and Y: Landmark coordinates normalized to [0.0, 1.0] by the image width and height, respectively.
  - Z: Represents the landmark depth with the depth at the midpoint of hips being the origin; the smaller the value, the closer the landmark is to the camera. The magnitude of Z uses roughly the same scale as X.
  - Visibility: A value in [0.0, 1.0] indicating the likelihood of the landmark being visible (present and not occluded) in the image.
2. Derive three regions of interest (ROIs) crops for each hand and the face, using the inferred pose landmarks. And employ a re-crop model to improve the ROI.
3. Crop the full-resolution input frame to these ROIs and apply task-specific face and hand models to estimate their corresponding landmarks.
4. Finally, merge all landmarks with those of the pose model to yield the full 540+ landmarks.

### 7.3. Data Augmentation

The dataset, comprising 16,000 videos, is duplicated by taking the reverse direction of each video, and then concatenating the two directions, resulting in a total of 32,000 videos.

### 7.4. Classification

The augmented dataset is divided randomly into three groups: 80% for training, 10% for testing, and 10% for validation. Due to restriction-free recording of the dataset, the KSU-SSL dataset is hard to analyze. The signers’ hands are blurry and difficult to detect and track most of the time. Thus, we built a BiLSTM model for classification. We chose LSTM specifically to classify words that consist of more than one sign. In addition, we chose BiLSTM to enhance the model’s performance, which runs the inputs in two ways, one from past to future and one from future to past. To avoid overfitting and enhance the model generalization on test data, we applied 50% dropout and batch normalization after each BiLSTM layer. In addition, two model architectures were evaluated on the dataset. The BiLSTM model has been trained once with two layers and once with three layers. Table 6 and Table 7 illustrate the applied BiLSTM architectures.

To minimize the estimated magnitude of learning error, an optimization function is applied to the network model. We have chosen the Adam optimizer, since it is a popular algorithm in the field of deep learning and because it achieves good results fast, especially in computer vision. The used learning rate in this model is 0.001. Different batch sizes have been used during the training of the data, including 32, 64, 128, and 512. In this model, the data have been trained on different numbers of epochs: once on 100 epochs, then on 200, 300, 500, and 600 epochs. Whenever we increase the number of epochs, the accuracy improves.

### 7.5. Model Evaluation Results

This section presents the model testing results. First, we must mention the environmental setup we have used to carry out this experiment. Python 3.7 was used to train the algorithm on a personal computer. The training was on variant models with different numbers of layers, batch sizes, epochs, and with data augmentation and without. The best accuracy was obtained with the three-layer model with data augmentation, 512 batch size, and 600 epochs, which took 10 h for extracting the features and 2 h for classification on a Google Colab pro+. Since Google Colab pro+ provides faster GPUs, we took a large number of batches. Other models were trained on Google Colab. Table 8 shows the results of the seven proposed modes.

To evaluate the model performance, several evaluation metrics play an essential role in evaluating the obtained testing result. We evaluated the performance of the seventh model (with the highest accuracy) using the precision, recall, F-1 score, and accuracy metrics. In addition, we used the Frame Per Second (FPS) rate.

**Figure 12:**
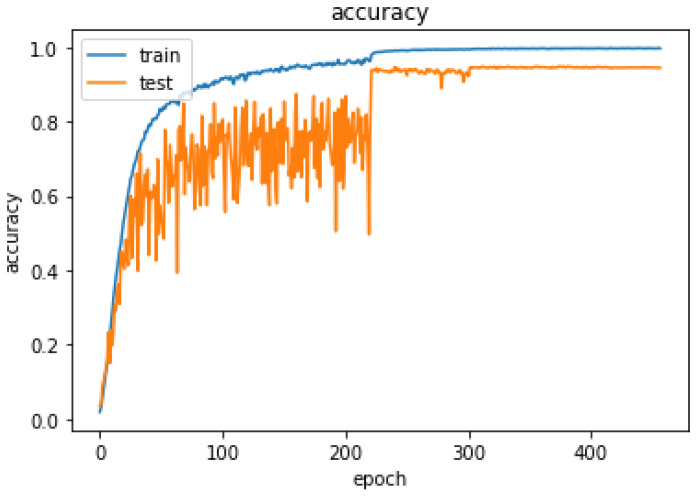
Performance of the model.

**Figure 13:**
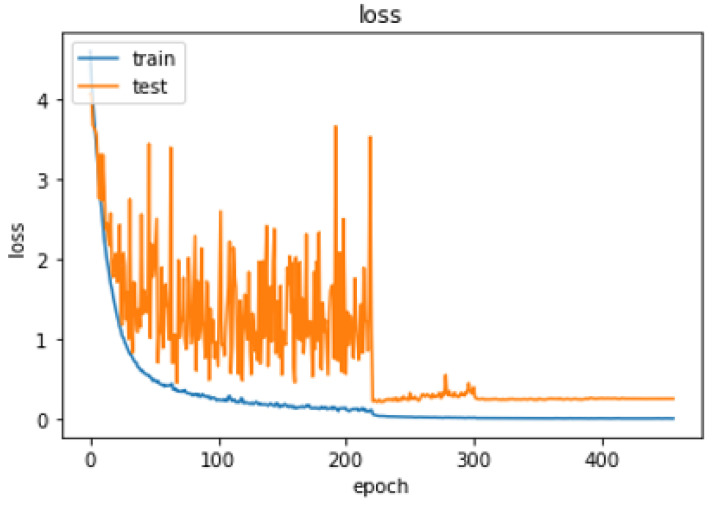
Loss behavior of the model.

Our model obtained 99.98% accuracy in training, 94.46% in testing, and 94.79% in validation. Figure 12 shows the accuracy of the proposed model and Figure 13 shows the loss behavior during training.

**Figure 14:**
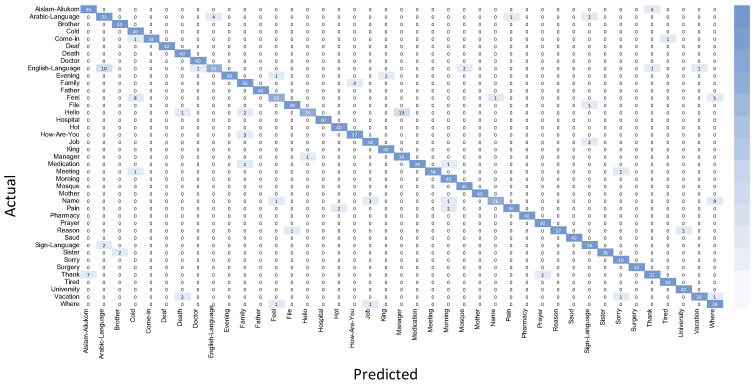
Confusion matrix.

Instead of measuring the total number of correct predictions (i.e., accuracy), it is better to measure the total number of positive and negative predictions. That is what the confusion matrix achieves. The confusion matrix is a table that illustrates the complete performance of the model concerning the test data by counting the number of true positives, true negatives, false positives, and false negatives, as represented in Figure 14.

The results of the best model are as follows. The precision is 94.61%, the Recall is 94.56%, and the F1-score is 94.52%. The translation process takes about 6 s on 30 frames. Thus, the FPS is calculated as follows: FPS = frames/seconds = 30/6 = 5.

The model has been tested using different types of participants (female, male, and children), with different hand features and movement speeds. The testing involved participants wearing different outfits, including women wearing veils and covering their faces. Moreover, the testing was carried out under different lighting conditions and various distances of signers from the mobile camera.

**Figure 15:**
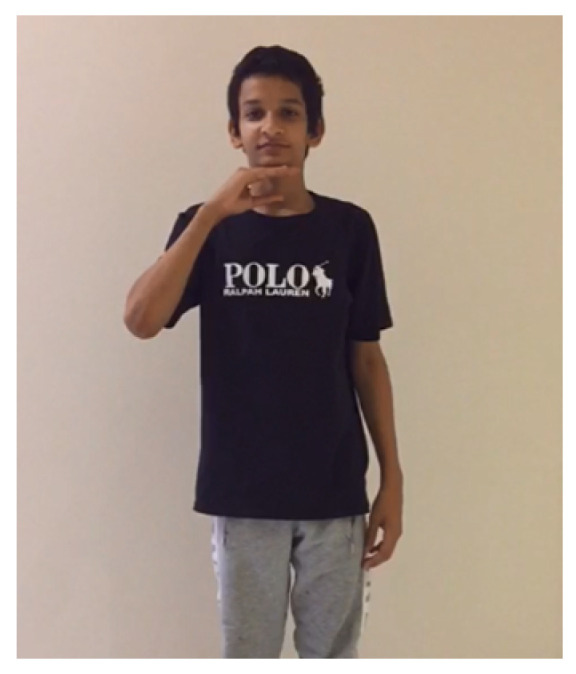
Father frame.

**Figure 16:**
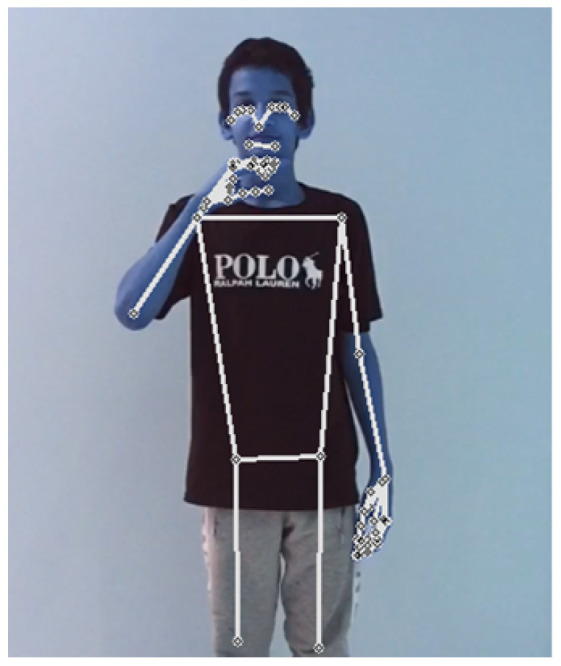
Father key points.

In what follows, we show two examples of the translation process on recorded videos, which our model has correctly classified. A video of a signer performing the sign “father”, as shown in Figure 15, was fed into the model. Firstly, the model extracted the key points as features, as illustrated in Figure 16. Secondly, the model correctly classified the performed sign as father. The process and feedback took a total of 20 s. The 20-second total translation includes the sign language translation time of 6 seconds, in addition to the overhead time for sending the video to the server and receiving the translated text back via an Internet connection.

**Figure 17:**
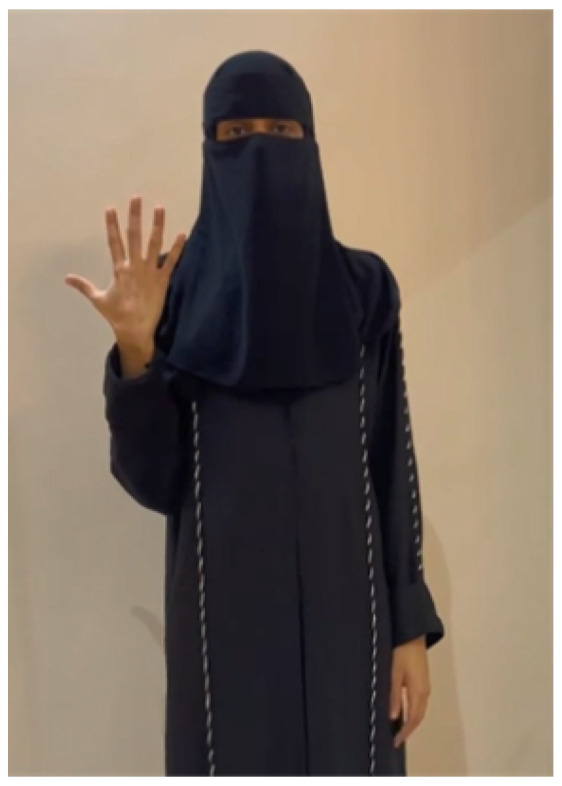
Five frame.

**Figure 18:**
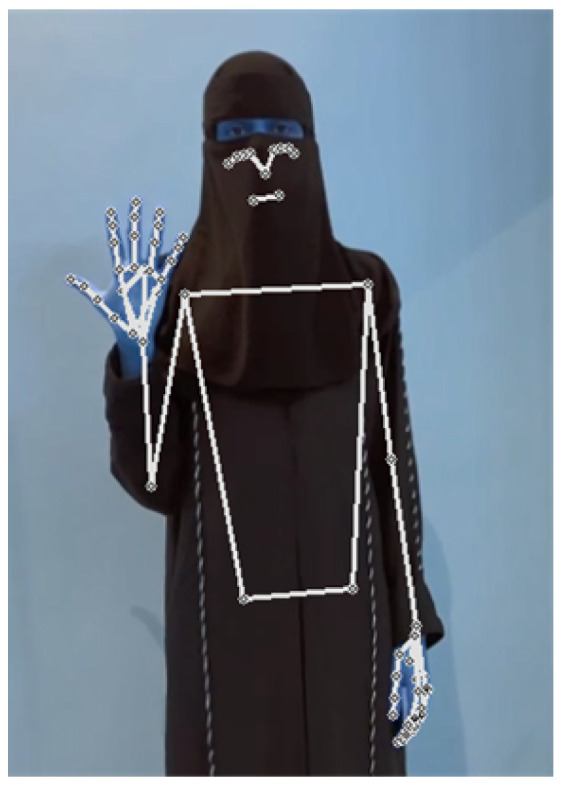
Five key points.

For the second example, a video of a signer covering her hair and face performing the sign of the number “5”, as shown in Figure 17, was fed into the model. The model successfully extracted the key points as features, as depicted in Figure 18. After that, the signed number was classified correctly. The process and feedback took a total of 19 s.

The deep learning model is connected to the Flutter application using Flask, which is an API created specifically to connect programs. First, the method takes a new video as input, then the video is pre-processed, and then the model predicts the sign. After that, the translated text is sent to the Flutter application. For translating from text to audio, we used a package in Flutter https://pub.dev/packages/flutter_tts (accessed on 10 May 2024), so the code is on the Flutter application and does not require an API.

## 8. Efhamni Mobile Application Testing

Software testing is an important part of the software development life cycle. The purpose of software testing is to assess software functionality to detect any system flaws and problems. Software testing seeks to improve program quality as much as possible by evaluating and validating the software code. In this paper, different testing approaches were utilized to assess the developed mobile application Efhamni. First, system testing was performed to verify that the integrated system components and functions were working as expected. Then, a heuristic evaluation was carried out to find any major flaws or problems that might hinder the usability of Efhamni. Finally, usability testing was carried out to assess the ease of use of Efhamni by potential users.

### 8.1. System Testing

System testing verifies the computer-based system’s completion and integration. It includes performing tests to evaluate the software code. The goal of testing the system is to guarantee that the program is functioning as expected. It also examines how the system’s components interact with one another to achieve the intended goals. System testing is mostly a black-box method of testing. The input is given to the system and the output is confirmed via black-box testing, which does not require any expertise or knowledge of the internal system structure or code [38]. Different test cases were identified and evaluated. Table 9 and Table 10 show the testing results of two out of twenty test cases performed on Efhamni.

### 8.2. Heuristic Evaluation

In heuristic evaluations, experts examine the design of an application or prototype against well-known usability principles or rules of thumb. Experts can be specialized in usability-related domains including cognitive psychology and human–computer interaction, interaction design, and user experience. It is preferred that the experts have previous expertise in the field and in reviewing similar products. Experts apply their knowledge about usability principles and expertise in the design field to evaluate the design. Typically, usability experts go through the software and put themselves in the place of a typical user to identify potential problems in the design. Heuristic evaluation is generally used before user testing to identify major interface and interaction issues and suggest modifications to improve the product usability. In this study, our proposed application, Efhamni, has been evaluated by two usability experts, who have evaluated the interface of the application using Nielsen’s User Interface Heuristics. The results of the heuristic evaluation of Efhamni are shown in Table 11.

### 8.3. Usability Testing

Usability testing is used to see if an application meets the needs of its users. The application’s testing is primarily focused on the application’s efficiency, tasks’ simplicity, ease of use and overall satisfaction with the application.

During the usability testing, users are observed while doing specific tasks and their opinions and reactions toward the application are recorded. Several issues and recommendations are discovered as a result of assessing the observations [38]. Objective and subjective measures are collected to evaluate the design and usability of the application developed in this study. Three objective measurements were used, which are task completion rate, time taken to complete each task, and the number of clicks. The subjective measures were used to record the user’s feelings and experiences during the testing process. Personal thoughts and beliefs also have an impact on this metric [38]. The subjective measurements were collected by filling out a post-test questionnaire that assessed the users’ ability to collaborate with other users while using the system, aesthetic design aspects related to the text, colors, icons, and photos, and the overall application simplicity and ease of use.

#### 8.3.1. Usability Testing Description

The usability testing of the Efhamni application was carried out by the design team on an Android mobile device. The usability testing included three deaf and hard-of-hearing participants and three hearing participants. The two groups have the same level of experience, and they were exposed to the same testing environment, tasks, and questions. Before starting the testing session, participants were asked to sign an informed consent form. The consent form explains to participants the purpose of the usability testing, how the data will be used, and their rights regarding the anonymity of the testing results and withdrawing from the test at any time if they are not willing to continue. Because this study involves some participants with disabilities (deaf and hard-of-hearing), the consent form signing was extremely important to ensure the privacy of the participants. A sign language facilitator helped to explain the test procedure and the participants’ rights.

During the usability testing session of Efhamni, each participant was asked to execute eleven tasks. The tasks have been selected carefully to test the main functionalities of the application. The tasks are as follows:

- Task 1: Create a new account;
- Task 2: Sign in;
- Task 3: Open ’How To Use’ interface;
- Task 4: Open information interface;
- Task 5: Record a video of the user performing sign language, edit the translation, and play the audio translation;
- Task 6: Import a video of the user performing sign language, edit the translation, and play the audio translation;
- Task 7: Open a chat interface;
- Task 8: Chat with a registered user;
- Task 9: Find the QR;
- Task 10: Open setting interface;
- Task 11: Sign out.

Task success relies on two parameters: completing the tasks successfully in the specified time and the number of clicks. Table 12 shows the acceptable ranges and numbers for both parameters, completion duration, and the number of clicks.

At the end of the user testing session, each participant has been requested to answer the post-test questionnaire questions as follows:

1. Did you find the application: easy to use, hard to use, or moderate?
2. Did you find the font size in the application: small, appropriate, or big?
3. Did you find the colors of the application: convenient, good, or unpleasant?
4. Did you find the placement of buttons on the screen: good, or need modification?
5. Did you find the wording of the application: appropriate, somewhat confusing, or unclear?
6. Is the application easy for daily use? yes, no.
7. Would you use the application in the future? yes, no.
8. Would you recommend the application to a friend: yes, maybe, no.
9. Open question about the user’s experience.

#### 8.3.2. Usability Testing Results and Analysis

Efhamni interfaces. Acceptable performance for task completion time occurs if the average task completion duration is within the specified range. Unacceptable performance for task completion time occurs if the average task completion duration is above the specified range. Acceptable performance for the number of clicks is reported if the average number of clicks is less than or equal to the specified number of clicks. Unacceptable performance for the number of clicks is reported if the average number of clicks is greater than the specified number of clicks. Unacceptable performance for ant task indicates issues that need to be addressed by the design team. Table 13 presents the average duration for completing each task by the six participants.

Table 14 summarizes the average number of clicks for completing each task by the six participants. All of the tasks’ results were within the acceptable ranges except Task 9. The average completion time for Task 9 was above the acceptable time duration. In addition, the average number of clicks for Task 9 was above the acceptable number. The reason behind this unacceptable performance was that the QR icon was not clear enough for the users. Therefore, the QR icon design has been updated to make it more visible and recognizable to users.

During the usability testing, several issues that the participants faced have been reported. For the first task, the deaf and hard-of-hearing users got confused between the creating a new account and signing in buttons. For Task 3, one of the users could not recognize the sidebar icon. For Task 5, two issues were reported. The first issue is that there were no instructions to guide the user before recording a video. The second issue is related to editing the translated text, where two users pressed on the text itself rather than the edit icon. For Task 9, the QR icon was not clear enough to users.

Table 15 lists the participants’ answers to the questions of the post-test questionnaire. Most of the participants found the application easy to use. All of them found the font size to be appropriate, the colors to be convenient, and the placement of buttons to be good. Also, they all agreed that the application is easy for daily use and they would use it in the future and would recommend it to a friend. The deaf and hard-of-hearing participants suggested including a library of videos for commonly used phrases performed in SSL.

Overall, the results of the usability testing revealed that the design of Efhamni was good and well-accepted by the target users. Based on the results and analysis of the usability testing, tasks which have not achieved an acceptable performance were fixed and rectified.

## 9. Discussion

Efhamni can be considered as the first mobile application to offer a unique set of features aimed at helping Saudi deaf and hard-of-hearing people to communicate easily with others using sign language without the need for a human translator. Its capabilities include detecting SSL from videos, whether they are recorded directly or uploaded, and providing both written and spoken translations of the SSL in the videos, in addition to the chat feature between the users of the application as well as the library in which users can save their videos for fast future sign language translation, thus bridging the communication gap for users.

Regarding the models used, most of the approaches considered a CNN variant, as they provided the best performance and are widely used in such applications. For instance, approaches that have considered utilizing CNN for ArSL/SSL are [3,10,11,12], and for non-ArSL, approaches are [23,35]. Therefore, we have chosen to use CNN in our system. Similar to the approaches proposed in [23,30,31,36], our application employed advanced technology, utilizing Pose Estimation combined with a BiLSTM architecture, which is usually used for sequence input to enhance the effectiveness and efficiency.

Notably, Efhamni achieves an accuracy level that is comparable to state-of-the-art approaches in this field. Moreover, to minimize delayed sign classification, it is critical to find a balance between accuracy and speed [11]. Therefore, our system is able to work in dynamic environments using a standard phone camera providing automatic translation. Additionally, our system fosters user interaction by allowing two registered users to engage in chat, enhancing its utility as a communication tool. Another convenient feature is the ability for users to save videos in their personal libraries, ensuring faster retrieval and access to frequently used or important sign language content. These innovative features collectively make Efhamni a pioneering tool in the realm of sign language translation and communication.

The KSU-SSL dataset contains 80 signs including the Arabic alphabet and numbers. The questionnaires were hard to share with the targeted group, but the results were satisfying and helpful. All three interviews added their own value, but confirmed the same facts obtained from each one. As for the requirements, the functional and non-functional requirements were gathered from the questionnaire and interviews. Moreover, the use case diagram and its description were presented. Finally, several testing methods were performed on the system.

The research implications of the Efhamni mobile application are significant, particularly for the Saudi deaf and hard-of-hearing community. This application not only serves deaf and hard-of-hearing individuals but also extends its utility to SL translators, interpreters, experts, and educators involved in teaching deaf and hard-of-hearing students. Additionally, it holds value for family members of the deaf and hard-of-hearing, offering them a tool to facilitate better communication. Audiologists may also find the application beneficial as part of their toolkit for patient interaction and support. Beyond individual use, the Efhamni application presents substantial implications for broader societal integration. Companies, healthcare facilities, and government entities aiming to support and effectively communicate with employees or clients who are deaf and hard-of-hearing can leverage this application. This suggests a wider scope for research into how such digital tools can enhance inclusivity and accessibility in various professional and public domains, thereby fostering a more accommodating environment for the deaf and hard-of-hearing community.

In our system Efhamni, we used MediaPipe, which is an open-source human pose estimation framework for feature extraction, which extracts key points as a skeleton using variant CNN models, such as MobileNetV2 for pose estimation, face detection, and Single-Short Detector (SSD) for hands. Since many of MediaPipe’s current solutions are designed with certain tasks in mind and are optimized for performance on those tasks without necessarily utilizing attention mechanisms, it is possible that these solutions do not explicitly require attention mechanisms. While adding attention mechanisms to MediaPipe might increase accuracy, attention mechanisms generally raise the computational costs of the model, which could result in slower inference and longer training times, particularly when the model is used on devices with limited resources, like mobile devices, which is the case with our system.

In this paper, we have focused on the translation of isolated SSL and we are aiming in the future to consider continuous sign language recognition for SSL. Although the application focuses on translating words rather than phrases, it is still considered beneficial and serves as an aiding tool for automatic translation as opposed to tools that rely on offline translators. Furthermore, isolated SL translation can be effective in circumstances when specific information must be given rapidly without using whole phrases. Furthermore, in emergency situations where necessary communication is required, such as in medical settings, the ability to translate critical signs can save lives or dramatically improve the quality of interaction between healthcare workers and patients, as is the case with our application. Although isolated sign language translation systems do not support entire sentences, they can help in enhancing sign language recognition technologies, since understanding and accurately translating individual signs is critical for constructing more complicated systems capable of handling phrases and sentences in the future.

Several key limitations are highlighted regarding the system’s capabilities. Like most of the available systems for sign language recognition, our proposed system, Efhamni, relies on limited corpora, with few video clips captured [39]. Using a limited corpus for sign language recognition can have several limitations that affect the performance of a sign recognition system. First, a smaller corpus which includes a small number of words or vocabulary may only identify a limited number of signs. Second, model training on a limited corpus increases the possibility of overfitting, in which the model performs well on training data but poorly on unseen data. Third, a limited corpus may not capture the diversity of sign language users, including differences in signing technique, pace, and physical traits (such as hand shapes and sizes). This lack of variety might result in less accuracy when the system is utilized by people whose signing style or physical attributes differ from those in the training set [40]. Fourth, sign language recognition depends heavily on the context and facial gestures to communicate information. A small corpus may not capture contexts or other features, such as facial expressions and body positions, which might affect the recognition accuracy. Fifth, most of the available corpora are domain-specific and cannot be generalized for all domains [40].

To improve the performance of sign language recognition systems and support real-time and practical applications, having a larger and more diverse dataset is required. This large corpus of varied sign language should contain thousands of video clips, using many signers with different features performing sign language gestures in a range of settings and contexts. However, collecting and annotating these data can be resource-intensive in terms of time and labor. Moreover, a major challenge associated with real-time sign language translation can be attributed to the lack of continuous sign language corpora. Sign language translation applications that are trained on isolated sign language corpora can translate words as opposed to phrases or sentences.

Another limitation is that the system is currently designed to interpret words individually rather than phrases or sentences. This means that to translate a complete sentence, the user would need to record and input each word separately, which could be time-consuming and may affect the fluidity and naturalness of communication.

The foundational elements of Efhamni have been successfully established, yet there remains considerable room for enhancement and development. Looking towards the future, several key areas have been identified for potential improvement. Firstly, expanding the dataset is crucial; by building a dataset that encompasses a broader scope, the system can cover a more extensive range of inputs and scenarios. Secondly, there is an opportunity to enhance the accuracy of the detection model. This can be achieved by employing advanced deep learning techniques, which could significantly refine the system’s ability to interpret and process information. Additionally, training the detection model on an alternative dataset could lead to improved performance, offering a more robust and reliable system. Finally, incorporating adjustable interface settings would empower users by allowing them to tailor the system’s interface to their preferences. This user-centric approach not only enhances usability but also ensures that Efhamni caters to a diverse range of needs and preferences.

## 10. Conclusions

The Saudi deaf and hard-of-hearing people require a lot of attention concerning their communication with the rest of the community. In this paper, we presented the design of a mobile application that facilitates communication between deaf and hard-of-hearing people and the rest of their communities by translating the SSL to audio and text using deep learning techniques in an online direct manner. Specifically, the system uses a CNN-BiLSTM model that translates SSL to Arabic text and audio. The system’s architecture and design were demonstrated using different diagrams, such as the use case, UML, sequence, and flowchart diagrams. A prototype of a mobile application, named Efhamni, was also developed to prove the applicability of the application idea. The implementation details were described and the prototype interfaces were presented. The deep learning model was evaluated and the results of our proposed model were presented. The developed mobile application’s functionalities were tested using black-box testing. In addition, heuristic evaluation with usability experts was employed where evaluators examined the interface and judged its compliance with usability principles. Based on the identified usability issues, the system interfaces were redesigned. Subsequently, usability testing on deaf and hard-of-hearing users, using objective and subjective measures, for the application’s most important functionalities was performed. The results show that the application is generally well-received by the target users and it was easy and effective to use.

## Figures and Tables

**Table 1:** Comparison of sign language datasets.

Dataset	No. of Samples	No. of Gestures	No. of Signers	Availability
AUTSL [23]	38,336	226	43	Yes
WLASL [24]	34,404	3126	119	Yes
HaGRID [25]	554,800	18	37,583	Yes
TheRuSLan [26]	10,660	164	13	No
SLOVO [27]	20,400	1000	194	Yes
ArSL [28]	3450	23	3	No
KSU-SSL [10]	16,000	80	40	No

**Table 2:** A comparison of state-of-the-art SL-to-text/audio approaches.

Reference	Year	Dataset	Approach	System Type	Evaluation (Accuracy)	Language
[12]	2023	KSU_SSL (293 images of Saudi Signs)	3DGCN	Desktop/ portable devices	97.25%	Saudi
[11]	2021	Arabic dataset (400 static and dynamic signs)	2DCNN, SKN	-	88.09–89.62%	Arabic
[3]	2020	Arabic dataset 40 Saudi Signs (27,301 static images )	CNN	Mobile device, Desktop	97.69–99.47%	Arabic
[10]	2020	KSU-SSL, ArSL RVL-SLLL	3DCNN	-	70–98.12%	Arabic
[36]	2020	ArSL	DeepLabv3+, CSOM, BiLSTM	-	89.5%	Arabic
[29]	2023	AUTSL 226 sign (38,336 samples)	MViT-SLR	-	95.72%	Turkish
[30]	2023	AUTSL, LRW	STF + LSTM 2DCNN, BiLSTM, ResNet, MLF	Mobile device	98.56%, 98.76%	Turkish
[31]	2023	AUTSL 226 sign (38,336 samples)	FE + LSTM	Mobile device	93.38%	Turkish
[32]	2022	AUTSL 226 sign (38,336 samples)	Ensemble - NTIS	-	96.37%	Turkish
[33]	2021	AUTSL 226 sign (38,336 samples)	VTN-PF	Desktop	92.92%	Turkish
[34]	2021	AUTSL 226 sign (38,336 samples)	SAM-SLR (RGB-D)	-	98.53%	Turkish
[23]	2020	AUTSL 226 sign (38,336 samples)	CNN + FPM + BLSTM + Attention	Desktop	62.02%	Turkish
[35]	2020	MNIST ASL dataset, 7172 images	CNN	-	93%	American

**Table 3:** Related applications.

Logo	Application	Language	Translation Direction	Dataset (Scope)	Platform	Accuracy	Features
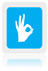	الترجمان Turjuman	Arabic (Standard)	Audio and text to ArSL (animated avatar)	Arabic words and letters, and numbers	IOS and android	- Text is accurate for words in the scope, if outside it is signed as letters. -Audio did not work.	Change avatar, express emotions, Subtitle speed control.
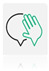	رمز Ramz	Arabic (Standard)	Audio and text to ArSL (animated avatar)	Arabic words and letters, and numbers	IOS	-Text is accurate for words in the scope, if outside it is signed as letters. -Audio did not work.	Export signed video, replay sign.
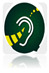	إحسان إنسان Ehsan Ensan	Arabic (Standard)	Audio and text to ArSL (animated avatar)	Arabic words and letters, and numbers	Android	-The app is not working at all.	Change avatar, Subtitle speed control, Chat.
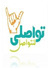	تواصلي Tawasuli	Arabic (Standard)	Audio, video, and text to ArSL(hand gesture icon) and vice versa	Arabic words and letters, and numbers	Android	-Text is signed as letters. -Audio and video did not work. -Hand gesture icon keyboard is typing, but translation in this direction does not work.	-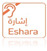	إشارة Eshara	Arabic (Standard)	Human sign language translator (Video call)	Arabic words and letters, and numbers	IOS and android	-The app has limited working hours (it does not work all the time)	-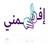	إفهمني Efhamni	Arabic (Saudi)	ArSL to text and audio (Video)	Arabic letters, numbers, and words (from the KSU- SSL dataset)	Android	-Effectively translates SSL into Arabic text and audio.	Chat between users, Ability to upload videos/images from camera roll.

**Table 4:** Experts questionnaire: questions and results.

Questions	Answers	Responses
What is your gender?	Male	70%
	Female	30%
What is your age group?	18–29	0%
	30–39	40%
	40–49	30%
	50+	30%
What is your education level?	Bachelor	50%
	Masters	40%
	PhD	10%
Do you interact with deaf people regularly?	Yes	100%
	No	0%
If your answer to the above question is Yes, for how long have you been interacting with deaf people?	1–3 years	0%
	4–7 years	20%
	8+ years	80%
According to your expertise, how well can Deaf people use Mobile Phones?	Expert	80%
	Moderate Experience	20%
	Novice	0%
According to your expertise, which communication method do deaf people use inside their homes?	Mobile application	0%
	Sign language	50%
	Writing	40%
	Other	10%
According to your expertise, which communication method do deaf people use outside their homes?	Mobile application	0%
	Sign language	60%
	Writing	40%
	Other	0%
According to your expertise, do deaf people face any difficulties when communicating with people in public places?	Yes	100%
	No	0%
If your answer to the above question is Yes, in which places do deaf people mostly face difficulties?	Hospitals	25%
	Government Offices	25%
	Malls	34%
	Restaurants	8%
	Other	8%
According to your expertise, what bothers deaf people the most when communicating with others in public places?	Inability to communicate	80%
	Feeling uncomfortable to ask	20%
	Other	0%
According to your expertise, do deaf people use applications to translate sign language?	Yes	40%
	No	60%
If your answer to the above question is Yes, what is the application?	Turjuman	35%
	Esharati	23%
	Eshara	42%
	Tawasuli	0%
	Other	0%
Would deaf people use an application to translate sign language to help them communicate in public places?	Yes	70%
	No	30%
Which feature is the most important in an application that translates sign language to text and audio?	Fast translation	55%
	Ease of Use	45%

**Table 5:** Deaf people questionnaire: questions and results.

Questions	Answers	Responses
What is your gender?	Male	5%
	Female	95%
What is your age group?	18–29	40%
	30–39	21%
	40–49	29%
	50+	10%
What is your education level?	Elementary School	12%
	Middle School	16%
	High School	39%
	Bachelor	30%
	Masters	0%
	PhD	3%
Are you deaf or do you have any hearing difficulty?	Yes	100%
	No	0%
How do you rate your level of experience in using mobile phones?	Expert	29%
	Moderate Experience	68%
	Novice	3%
How many hours do you spend using your phone daily?	0-2 h	6%
	3-5 h	73%
	6+ hours	21%
Which communication method do you use when communicating with others inside your home?	Mobile application	0%
	Sign language	69%
	Writing	22%
	Other	9%
Which communication method do you use when communicating with others outside your home?	Mobile application	13%
	Sign language	44%
	Writing	36%
	Other	7%
Do you face any difficulty when communicating with people in public places?	Yes	61%
	No	39%
If your answer to the above question is Yes, where do you mostly face these difficulties?	Hospitals	39%
	Government Offices	14%
	Malls	16%
	Restaurants	15%
	Other	16%
What bothers you the most when communicating with others in public places?	Inability to communicate	60%
	Feeling uncomfortable to ask	37%
	Other	3%
Have you ever used an application for sign language translation?	Yes	54%
	No	46%
If your answer to the above question is Yes, what is the application?	Turjuman	25%
	Esharati	27%
	Eshara	30%
	Tawasuli	16%
	Other	2%
Would you use an application to translate Sign Language to help you communicate in public places?	Yes	58%
	No	42%
Which feature is the most important in an application that translates sign language to text and audio?	Fast translation	46%
	Ease of Use	54%

**Table 6:** Two-layer BiLSTM architecture.

Bidirectional (LSTM (128, activation = ’tanh’))
Dropout (0.5)
BatchNormalization ()
Bidirectional (LSTM (128, activation = ’tanh’))
Dropout (0.5)
BatchNormalization ()
Dense (128, activation = ’tanh’)
Dropout (0.5)
BatchNormalization ()
Dense (activation = ’softmax’)

**Table 7:** Three-layer BiLSTM architecture.

Bidirectional (LSTM (128, activation = ’tanh’))
Dropout (0.5)
BatchNormalization ()
Bidirectional (LSTM (128, activation = ’tanh’))
Dropout (0.5)
BatchNormalization ()
Bidirectional (LSTM (256, activation = ’tanh’))
Dropout (0.5)
BatchNormalization ()
Dense (128, activation = ’tanh’)
Dropout (0.5)
BatchNormalization ()
Dense (activation = ’softmax’)

**Table 8:** Recognition accuracy (%) achieved with different models.

Proposed Model	Number of Layers	Batch Size	Epochs	Data Augmentation	Classification Time	Accuracy
1st	3	32	100	No	5 h	79.62
2nd	2	32	100	No	3 h	87.06
3rd	2	64	200	No	4 h	93.06
4th	2	64	200	Yes	12 h	80.46
5th	2	64	500	No	1 h	93.37
6th	2	128	300	No	4 h	92.75
7th	3	512	600	Yes	2 h	94.46

**Table 9:** Translation of sign language to text and audio.

Test Case 4.2: Translate Sign Language to Text and Audio			
Description: User records a video performing Saudi sign language then translate it into text and audio			
Pre-conditions: User is in home page			
Test Case Title: User records video performing sign language gesture showing only hands.			
Test Steps:			
1. Opens application			
2. User logs in			
3. Clicks on “Record a video”			
4. Records video performing a sign language gesture			
5. Click “Translate” button			
6. Press edit icon			
7. Enters text in edit field			
8. Click “Edit” button			
9. Click audio icon			
**Test Data**	**Expected Results**	**Actual Results**	**Pass/Fail**
Records a video performing a sign language gesture showing only the hands	Recorded video is accepted and translated but if the translation is not accurate, the user can edit the translation and play audio back.	As expected	Pass

**Table 10:** Chat with another user.

Test Case 7.2: Chat with Another User			
Description: Two registered users chat with each other via email			
Pre-conditions: Logged in user			
Test Case Title: Search for an email that does not exist in the system			
Test Steps:			
1. Opens application			
2. Logs in			
3. Clicks on “Quick Chat” button			
4. Enters the email of the other registered user			
5. Click on “Search” button			
**Test Data**	**Expected Results**	**Actual Results**	**Pass/Fail**
Email: sara@gmail.com	Shows no results in the search bar	As expected	Pass

**Table 11:** Heuristic evaluation.

#	Criteria	Usability Test Experts Comments	Average Score of 10
**1**	Visibility of system status		10
**2**	Match between system and the real world		9.5
**3**	User control and freedom	User control is missing (e.g., the tab navigation between cells and there is no go back buttons among the interfaces).	6
**4**	Consistency and standards	Translation interface needs color modification.	9.5
**5**	Error prevention	Show the user the password restrictions.	9.5
**6**	Recognition rather than recall		6.5
**7**	Flexibility and efficiency of use	The phrase “and” is not clear.	6
		Make Sign out button more obvious.	
**8**	Aesthetic and minimalist design		10
**9**	Help users recognize and diagnose and recover from errors	Password error and the error message should be shown once the user makes a mistake.	7.5
**10**	Help and documentation		10

**Table 12:** Tasks success measurement criteria.

Tasks	Completion Duration	Number of Clicks
Task 1	50–65 s	9
Task 2	20–30 s	4
Task 3	10–20 s	2
Task 4	1–3 s	1
Task 5	30–40 s	5
Task 6	3–15 s	3
Task 7	5–10 s	1
Task 8	2–12 s	1
Task 9	3–5 s	2
Task 10	5–10 s	2
Task 11	2–6 s	3

**Table 13:** Summary of average completion time.

Tasks	Average Completion Duration	Task Performance
Task 1	56 s	Acceptable
Task 2	17 s	Acceptable
Task 3	16 s	Acceptable
Task 4	3 s	Acceptable
Task 5	40 s	Acceptable
Task 6	9 s	Acceptable
Task 7	16 s	Acceptable
Task 8	3 s	Acceptable
Task 9	17 s	Unacceptable
Task 10	4 s	Acceptable
Task 11	2 s	Acceptable

**Table 14:** Summary of average number of clicks.

Tasks	Average Number of Clicks	Task Performance
Task 1	9	Acceptable
Task 2	4	Acceptable
Task 3	2	Acceptable
Task 4	1	Acceptable
Task 5	5	Acceptable
Task 6	3	Acceptable
Task 7	1	Acceptable
Task 8	1	Acceptable
Task 9	5	Unacceptable
Task 10	2	Acceptable
Task 11	3	Acceptable

**Table 15:** Post-test participant answers.

Participant	Q1	Q2	Q3	Q4	Q5	Q6	Q7	Q8
1	Moderate	Appropriate	Convenient	Good	Somewhat confusing	Yes	Yes	Yes
2	Easy	Appropriate	Convenient	Good	Appropriate	Yes	Yes	Yes
3	Easy	Appropriate	Convenient	Good	Appropriate	Yes	Yes	Yes
4	Moderate	Appropriate	Convenient	Good	Somewhat confusing	Yes	Yes	Yes
5	Easy	Appropriate	Convenient	Good	Somewhat confusing	Yes	Yes	Yes
6	Easy	Appropriate	Convenient	Good	Appropriate	Yes	Yes	Yes

## Data Availability

The data of this study, KSU-SSL, was obtained from King Saud University with authors’ permission. Access to this data is restricted, but interested researchers may contact Prof. Mansour Alsulaiman (msuliman@ksu.edu.sa) for inquiries regarding data access.
